# Comparative Genomics Provides Insights Into Genetic Diversity of *Clostridium tyrobutyricum* and Potential Implications for Late Blowing Defects in Cheese

**DOI:** 10.3389/fmicb.2022.889551

**Published:** 2022-06-02

**Authors:** Lucija Podrzaj, Johanna Burtscher, Konrad J. Domig

**Affiliations:** Department of Food Science and Technology, Institute of Food Science, University of Natural Resources and Life Sciences, Vienna, Austria

**Keywords:** *Clostridium tyrobutyricum*, spoilage, comparative genomics, pangenome, dairy, cheese

## Abstract

*Clostridium tyrobutyricum* has been recognized as the main cause of late blowing defects (LBD) in cheese leading to considerable economic losses for the dairy industry. Although differences in spoilage ability among strains of this species have been acknowledged, potential links to the genetic diversity and functional traits remain unknown. In the present study, we aimed to investigate and characterize genomic variation, pan-genomic diversity and key traits of *C. tyrobutyricum* by comparing the genomes of 28 strains. A comparative genomics analysis revealed an “open” pangenome comprising 9,748 genes and a core genome of 1,179 genes shared by all test strains. Among those core genes, the majority of genes encode proteins related to translation, ribosomal structure and biogenesis, energy production and conversion, and amino acid metabolism. A large part of the accessory genome is composed of sets of unique, strain-specific genes ranging from about 5 to more than 980 genes. Furthermore, functional analysis revealed several strain-specific genes related to replication, recombination and repair, cell wall, membrane and envelope biogenesis, and defense mechanisms that might facilitate survival under stressful environmental conditions. Phylogenomic analysis divided strains into two clades: clade I contained human, mud, and silage isolates, whereas clade II comprised cheese and milk isolates. Notably, these two groups of isolates showed differences in certain hypothetical proteins, transcriptional regulators and ABC transporters involved in resistance to oxidative stress. To the best of our knowledge, this is the first study to provide comparative genomics of *C. tyrobutyricum* strains related to LBD. Importantly, the findings presented in this study highlight the broad genetic diversity of *C. tyrobutyricum*, which might help us understand the diversity in spoilage potential of *C. tyrobutyricum* in cheese and provide some clues for further exploring the gene modules responsible for the spoilage ability of this species.

## Introduction

*Clostridium tyrobutyricum* is a Gram-positive, strictly anaerobic, spore-forming bacterium belonging to the genus *Clostridium* (Rainey et al., 2015). This species was first described by Van Beynum and Pette (1935), in which the prefix “tyros” refers to the cheese, the initial isolation source of the bacterium (Ivy and Wiedmann, 2014). Other isolation sources include silage, slurry, the human and animal intestine, and mud (Langó and Heinonen-Tanski, 1995; Heinonen-Tanski et al., 1998; Julien et al., 2008). *Clostridium tyrobutyricum* is characterized by intensive fermentation of carbohydrates to butyric acid and acetic acid as main products, as well as carbon dioxide (CO_2_) and hydrogen (H_2_) as side products. Simple medium requirements for cell growth, relatively high product purity and yield have made *C. tyrobutyricum* an organism of significant interest to different industries, including chemical and pharmaceutical industry (Michel-Savin et al., 1990; Jiang et al., 2010; Wu et al., 2017). In the dairy industry, however, butyric acid fermentation of *C. tyrobutyricum* in cheese often leads to severe quality defects of cheese and consequently considerable financial losses for dairy producers (Doyle et al., 2015; Brändle et al., 2016).

In light of the significance of *C. tyrobutyricum*, many methods have been applied to characterize this species, and considerable heterogeneity has been observed on species and strain level in terms of their genetic and phenotypic characteristics. In order to genetically characterize *C. tyrobutyricum* on the subspecies or strain level, several approaches have been used, including Matrix-Assisted Laser Desorption Ionization-Time Of Flight Mass Spectrometry (MALDI-TOF MS) typing (Burtscher et al., 2020), Multilocus variable-number of tandem repeat analysis (Nishihara et al., 2014), pulsed field gel electrophoresis (PFGE; Garde et al., 2012b), restriction fragment length polymorphism, and repetitive element palindromic PCR (rep-PCR; Bermudez et al., 2016; Burtscher et al., 2020). All these studies lead to the main conclusion of high intra-species diversity within *C. tyrobutyricum*. Accordingly, also phenotypical characterization showed high variation among the tested strains (Garde et al., 2012a; Ruusunen et al., 2012; Silvetti et al., 2018). In our recent study, the comparison of the phenotypes of 12 *C. tyrobutyricum* strains, which were selected from 157 strains on the basis of genotypic and proteotypic variability, revealed a high degree of heterogeneity considering the time until gas production was visible at different incubation temperatures in an experimental cheese broth (Podrzaj et al., 2020). This finding indicated that certain characteristics, such as the ability to produce gas and organic acids may be strain-dependent, highlighting the fact that the capability of *C. tyrobutyricum* to spoil cheese may be a strain-specific feature. However, despite these insights, understanding the genetic background and functional traits underpinning *C. tyrobutyricum* spoilage ability remain limited.

With the rapid development of next generation sequencing technology and bioinformatics, comparative genomics can provide a novel way to effectively assess the genetic diversity of bacteria (Zhou et al., 2014). Although numerous genomic comparative studies were performed on members of *Clostridium* Cluster *sensu stricto*, only a few are specifically concerned with *C. tyrobutyricum* (Schiefer-Ullrich et al., 1984; Zhou et al., 2014; Williamson et al., 2016; Palevich et al., 2021). For instance, whole-genome sequencing of *C. tyrobutyricum* has uncovered the genomic basis for their potential application in industry and human health (Liu et al., 2020). Based on comparative analysis within *C. tyrobutyricum* and within *Clostridium* spp., Storari et al. (2016) provided significant information about nutritional requirements. However, research to date is limited to a small number of *C. tyrobutyricum* strains (18 strains). Recently, several genomes of strains isolated from raw milk and cheese previously phenotypically characterized (Podrzaj et al., 2020), have been made available (Podrzaj et al., 2021), as well as strains from silage (Munier et al., 2019). These datasets have provided the opportunity for comparative studies that examine genetic diversity of *C. tyrobutyricum* associated with LBD in cheese.

In this study, we aimed to further elucidate the genetic diversity of *C. tyrobutyricum* and to evaluate strain-level differences by exploring genes in their gene repertoire that may contribute to strain-dependent spoilage potential. In addressing these aims, we performed comparative analysis on genomes of 28 *C. tyrobutyricum* strains available in the National Center for Biotechnology Information (NCBI) GenBank database.

## Materials and Methods

### Genomic Dataset of *Clostridium tyrobutyricum*

All available genomic sequences of *C. tyrobutyricum* with different assembly levels were downloaded from the NCBI GenBank database^1^ in October 2021, including three complete and 29 draft genomes. Metadata were also collected for each genome both from the NCBI GenBank database and the BioSample entries,^2^ which included, among others, information on the assembly, about the collection date, country, isolation source, and isolation year. We then investigated all accessible genome records of *C. tyrobutyricum* and if a strain was sequenced multiple times, we chose the assembly that had the highest level of genome completeness. After screening, 28 genomic sequences of *C. tyrobutyricum* remained. The final dataset comprised strains isolated from various sources, including cheese, raw milk, silage, mud, and human stool, and a strain with unknown source. Details of strains involved in the current study with respect to isolation data (collection date, source, isolation date, and country of origin) and genome assembly accession numbers are provided in Supplementary Table S1.

### Gene Predictions and Functional Annotations

To ensure the uniformity of the attributed genomic annotations, sequences of all *C. tyrobutyricum* genomes were analyzed using the same protocol. The protein-coding genes were predicted and re-annotated using Prokka suite v1.14.6 (parameters: -genus Clostridium -species tyrobutyricum -evalue 1e-09 -coverage 80 -mincontiglen 200; Seemann, 2014). Functional assignments were performed and manually edited based on similarity searches against the Clusters of Orthologous Groups (COGs) using eggNOG-mapper (Huerta-Cepas et al., 2019) and Kyoto Encyclopedia of Genes and Genomes (KEGG) Orthology And Links Annotation (KOALA; Kanehisa et al., 2016). Values associated with COG categories represented the percentage of COGs belonging to each category out of the total number of identified COGs. If a gene was assigned to two COG categories, each COG category was counted separately. CRISPR direct repeats (DRs) and spacers were identified using the online tool CRISPRCasFinder^3^ (Couvin et al., 2018).

### Comparative Genomics

Pairwise average nucleotide identity (ANI) values were determined using the python script PyANI v0.2.7,^4^ which estimates ANI based on BLAST+ (ANIb; Camacho et al., 2009) or MUMmer (ANIm; Kurtz et al., 2004). Heatmaps were also generated using PyANI to provide visualization of the level of identity between *C. tyrobutyricum* genomes. Digital DNA-DNA hybridization (dDDH) values were calculated utilizing the web tool Genome-to-Genome Distance Calculator GGDC^5^ using the recommended BLAST+ method (Meier-Kolthoff et al., 2013). The dDDH results were based on the recommended Formula 2 (identity/high-scoring segment pair length), which is independent of genome length and thus can serve as robust indicator for incomplete draft genomes. The thresholds for species demarcation were 95 and 70% for ANI and dDDH, respectively (Richter and Rossello-Mora, 2009).

### Construction of Core- and Pangenomes

Roary (Page et al., 2015) was utilized to calculate core- and pangenome of *C. tyrobutyricum* with identity cut-off of 95%. Four clusters of genes were defined as previously described by Kaas et al. (2012). Briefly, the core and soft-core genome represented the genes or the proteins identified in at least 95–99% of the strains, respectively. All genes present in more than two but less than 95% of the strains were classified as accessory genes, whereas genes exclusive to one strain were classified as strain-specific genes. Results were visualized by using the script “roary_plots.py.”^6^

The core- and pangenome profiles were obtained using the method proposed by Knight et al. (2016) and the models given by Tettelin and colleagues (Tettelin et al., 2005, 2008; Rasko et al., 2008). Briefly, curve fitting of the pangenome was performed using a power-law regression based on Heaps’ law ( $y_{\mathrm{pan}}=A_{\mathrm{pan}}x^{B\mathrm{pan}}+C_{\mathrm{pan}})$ , as previously described (Tettelin et al., 2005, 2008; Rasko et al., 2008) using the PanGP Software (Zhao et al., 2014). In the formula, y_pan_ is the pangenome size, x is the number of genomes considered, and A_pan_, B_pan_, and C_pan_ are fitting parameters. When 0 < B_pan_ < 1, the size of the pangenome increases unboundedly with sequential additions of new genomes, thus indicating an open pangenome. Conversely, when B_pan_ < 0 or B_pan_ > 1, the pangenome is considered closed since the trajectory approaches a constant as more genomes are considered. Number of core genes after addition of each new genome was plotted as a function of the number of genomes added sequentially, in similar manner as pangenome plot. The exponential curve fit model ( $y_{\mathrm{core}}=A_{\mathrm{core}}e^{\mathrm{core}\;x}+C_{\mathrm{core}}$ ) was used to fit the core genome (Tettelin et al., 2005, 2008; Rasko et al., 2008). Here, y_core_ denotes core genome size, x denotes number of genomes, A_core_, B_core_, and C_core_ are fitting parameters. Both the core and pangenomes were visualized through PanGP (Zhao et al., 2014) using the gene presence-absence binary matrix obtained from Roary as an input.

### Phylogenetic Analyses

The 16S rRNA gene sequences from all *C. tyrobutyricum* genomes were obtained from the NCBI GenBank database and used to query the EzTaxon server^7^ to identify their closest relatives. When a strain contained several copies of the same ribosomal RNA gene, we chose the longest gene to provide a single sequence for phylogenetic analysis. Phylogenetic analyses were further carried out by comparing query sequences along with those from relatives with >95% similarity namely, *Clostridium butyricum* ATCC 19398^T^, *Clostridium beijerinckii* JCM 1390^T^, *Clostridium sporogenes* DSM 795^T^, *Clostridium algifaecis* NCFB 2931, *Clostridium kluyveri* NBRC 12016, and *Clostridium ljungdahlii* DSM 13528. The 16S rRNA sequences were aligned with ClustalW (Thompson et al., 1994) and a maximum-likelihood tree using the Kimura 2-parameter model (Kimura, 1980) with 500 bootstrap replicates was constructed. These sequences were downloaded from the NCBI database.

The pangenome tree based on presence/absence of each gene family in all genomes was constructed using hierarchical clustering of the relative Manhattan distance according to the distance matrix generated by Roary. For core genome-based phylogeny, core gene alignment generated by Roary was used, and the phylogenetic tree was constructed using the maximum-likelihood algorithm with 500 bootstraps. Trees were generated using MEGA X v10.1 (Kumar et al., 2018).

To further analyze phylogenetic similarities among the 28 *C. tyrobutyricum* strains, we used single nucleotide polymorphisms (SNPs) data found in the core genes. The SNPs between the *C. tyrobutyricum* genomes were identified pairwise using kSNP3 program using a k-mer of size 17 (Gardner et al., 2015). This k-mer size of 17 nucleotides was chosen as this was the shortest k-mer that provided a uniqueness greater than 99% (k-mer 17 uniqueness = 99.1%, k-mer 15 uniqueness = 95.5%). Phylogenetic relationships among the 28 genomes were reconstructed by maximum likelihood (ML) using a set of core-SNPs detected.

## Results and Discussion

### General Genomic Features of *Clostridium tyrobutyricum*

General genome features of the 28 *C. tyrobutyricum* strains used in this study are presented in Supplementary Table S1. Briefly, the complete genomes ranged in size from ~3.07 to ~3.13 Mbp, whereas the draft genomes varied from ~2.63 to ~3.33 Mbp. According to these sizes, the numbers of coding sequences (CDSs) also varied among the complete genomes (2,945–3,015) and among the draft genomes (2,497–3,356), which is equivalent to an overall 26.9% difference. *Clostridium tyrobutyricum* genomes had a low overall guanine and cytosine content (GC content) showing only minor differences among strains varying from 30.2 to 31.1%. Major differences were observed for the number of rRNAs and tRNAs encoded, with fewer in all draft genomes than in complete genomes except for FAM22552 and FAM22553 (Supplementary Table S1). Because rRNAs, tRNAs, and other repetitive sequences are recognized as problematic regions in genome assembly, draft status may explain the discrepancy among the number of these noncoding sequences of *C. tyrobutyricum* strains between draft and complete genomes.

**Figure 1:**
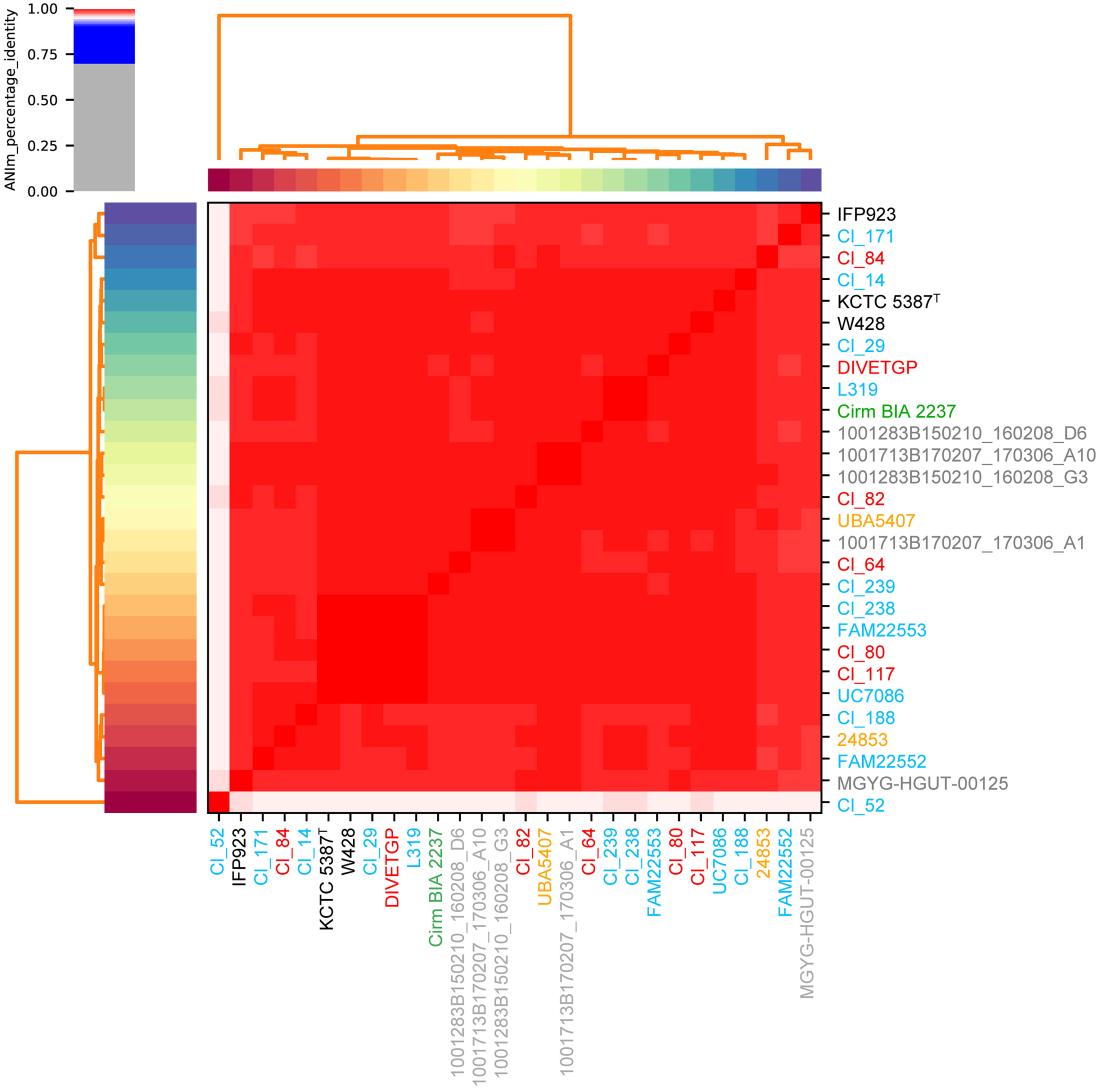
Heatmap of the average nucleotide identity (ANI) of 28 *Clostridium tyrobutyricum* strains. ANIm analysis based on MUMmer alignment (Kurtz et al., 2004) of the genome sequences was performed and visualized using pyANI (see Footnote 4). The strain names are colored according to their isolation source, where red, blue, green, gray, and orange denote milk, cheese, silage, human, and mud, respectively. The type strain is indicated by the superscript capital T.

In pairwise comparisons between all genomes except one (i.e., Cl_52) both ANI and dDDH values were higher than the corresponding cut-off point for species delineation (95 and 70%, respectively; Richter and Rossello-Mora, 2009), indicating that the strains were confirmed to belong to the species *C. tyrobutyricum* with only minor intra-species differences (Figure 1; Supplementary Tables S2–S4). As expected, the MUMmer-based whole-genome alignment revealed that the sequences of two strains, namely type strain KCTC 5387 and strain W428, aligned at a relatively high level with each other, showing minimal genomic rearrangements when compared with other *C. tyrobutyricum* strains (Supplementary Table S2). This observation is not surprising considering the fact that W248 derived from *C. tyrobutyricum* type strain, as previously described (Wu et al., 2017). However, in case of strain Cl_52, ANIm, ANIb, and dDDH values were on the borderline of the species threshold. Although ANIm and ANIb values between this strain and other test strains ranged from 95.02 to 95.38% and 94.68 to 95.26%, respectively, dDDH values were only between 60.2 and 62.5% [with 95% confidence (Supplementary Tables S3–S5)]. These results may indicate to some degree the genomic heterogeneity present among *C. tyrobutyricum* strains. In the light of more recent studies, a threshold of 94% ANI was also proposed as the putative boundary for intraspecies delineation, which corresponds to a lower dDDH range of ≈60–70% (Richter and Rossello-Mora, 2009; Meier-Kolthoff et al., 2013). However, given that the pairwise comparisons between all other 27 strains exhibited dDDH higher than 85.5%, the classification of Cl_52 as *C. tyrobutyricum* should be queried. It remains to be examined whether strain Cl_52 presents enough uniqueness from the phenotypic and genotypic standpoint to be considered a novel species different from, or subspecies within, *C. tyrobutyricum*.

### Phylogenetic and Phylogenomic Analysis

The comparison of almost complete 16S ribosomal RNA (16S rRNA) gene sequences is commonly used to establish taxonomic relationships between prokaryotic strains, with 98.65% similarity currently recognized as the cutoff for delineating species (Kim et al., 2014). Therefore, to determine the taxonomy and phylogenetic relatedness between the genomes of *C. tyrobutyricum* strains and a subset of closely related *Clostridium* species, we performed a comparative analysis of their 16S ribosomal RNA (16S rRNA) gene sequences (Supplementary Figure S1). *Clostridium tyrobutyricum* was clearly separated from other *Clostridium* species, with closest relatives identified as *C. ljungdahlii* and *C. kluyveri.* The 16S rRNA regions appear to be highly conserved (identity of 99.2–100%; Supplementary Table S6) across 27 of 28 *C. tyrobutyricum* strains, while the sequence similarity was lowest between strain Cl_52 and the other 27 *C. tyrobutyricum* strains. These results corroborate previous findings, where based on MALDI-TOF MS, hexaplex-PCR, and rep-PCR typing of *C. tyrobutyricum* strains, strain Cl_52 was clustered separately from all other test strains (Burtscher et al., 2020; Podrzaj et al., 2020). Given the fact that MALDI-TOF MS represents a phenotype based on ribosomal proteins and other abundant proteins in the bacterium, and 16S rRNA is a component of the ribosome, high concordance between the results of MALDI-TOF MS-based typing and 16S rRNA gene comparison may be expected. However, it should be noted that some *C. tyrobutyricum* strains present multiple copies of the 16S rRNA gene and that there may be variations among them. Thus, the choice of 16S rRNA genes may influence the structure of the 16S rRNA gene-based phylogenetic tree (Coenye and Vandamme, 2003). In fact, nine out of 28 genomes of *C. tyrobutyricum* contained more than one copy of the 16S rRNA gene (Supplementary Table S1). Hence, the phylogeny based on 16S rRNA genes or other ribosomal proteins may therefore not be applicable for the determination of relationships among *C. tyrobutyricum* strains due to the missing alignment information of other fundamental genes (Yu et al., 2019). Thus, their phylogenetic relationships need to be resolved using robust higher resolution analyses.

**Figure 2:**
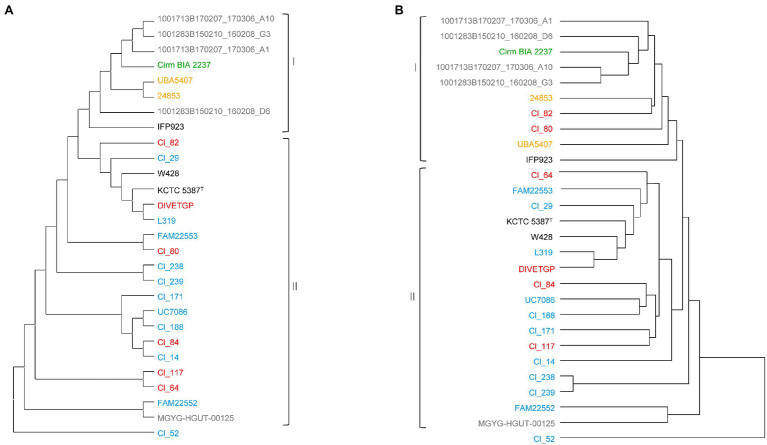
Phylogenetic analysis of 28 *Clostridium tyrobutyricum* strains. **(A)** Maximum-likelihood (ML) phylogenetic tree based on 1,179 single-copy gene families using 500 bootstrap replications. **(B)** Pangenome tree based on presence/absence of gene family in all genomes. The strain names are colored according to their isolation source, where red, blue, green, gray, and orange denote milk, cheese, silage, human, and mud, respectively. The type strain is indicated by the superscript capital T. The phylogenetic trees were visualized using MEGA X v10.1 (Kumar et al., 2018).

We next investigated the phylogenetic relationships among the test strains by constructing two phylogenetic trees: one based on the concatenated alignment of 1,179 single-copy core genes shared by the 28 *C. tyrobutyricum* strains (Figure 2A), and another based on Manhattan distance between the 28 *C. tyrobutyricum* strains, which segregated the strains on the basis of the absence or the presence of each gene family (Figure 2B). Overall, a large genomic heterogeneity was observed and strains isolated from the same or similar isolation sources, with these being mud, silage, human stool, raw milk and cheese, were dispersed across both trees (Figures 2A,B).

Despite the wide genome diversity, according to the topology in the two trees, we identified two main clades (clade I and clade II; Figure 2). In both trees, clade I was made out of all except one (MGYG-HGUT-00125) human isolates, one silage isolate (Cirm BIA 223799), two mud isolates (i.e., UBA5407 and 24853) and strain IFP923 with unknown isolation source. The second clade (clade II) consisted of genomes of the isolates from raw milk and cheese in addition to the type strain KCTC 5387, which also had been initially isolated from cheese, and its derivate (W428). However, there was discordance in the phylogenetic placement of two milk isolates, namely Cl_82 and Cl_80, between the two trees, as they dispersed away from other milk and cheese isolates in the pangenome tree and grouped together with silage, mud, and human isolates comprising clade I. Interestingly, these two strains were isolated from raw milk obtained from the same production location. It appears that the non-core genes made these two strains divergent from the rest of milk/cheese isolates.

It can be seen, that isolates from raw milk and cheese tend to cluster together (Figure 2). This observation is not surprising due to the fact that the spores that contaminate raw milk survive milk and cheese production processes. Occasionally, genomes of the isolates obtained from the same food source (i.e., cheese) clustered together under the same branch, e.g., UC7086 and Cl_188, and Cl_238 and Cl_239 suggesting that they are closely related. Indeed, Cl_238 and Cl_239 were isolated from the same cheese. Mostly, the clusters, which encompassed more than two strains, consisted of a mixture of isolates from different isolation sources. For instance, cheese isolate L319 clustered with strain DIVETGP isolated from raw milk in both trees, suggesting high similarity between their genetic contents. Furthermore, Cl_14 and Cl_84 co-clustered under one node in the core genome-based tree despite their different isolation sources (cheese and raw milk, respectively). However, they dispersed away from one another in the pangenome tree. We speculate that the variations in accessory genes of these two strains contribute to their differences. Similarly, cheese isolate FAM22553 showed close relatedness to milk isolate Cl_80 in the core genome-based tree, but exhibited substantial difference in the pangenome-based tree, indicating that genetic variation in non-core genes likely made the two strains divergent. Moreover, two strains, namely, Cl_171 and Cl_117, isolated from cheese and milk, respectively, clustered under same node in the pangenome tree, but showed no close relationship in the core genome-based tree. This may be due to the accessory genes that made up a dominant proportion of the phylogenetic signal and contributed to the evolution of these two strains.

It is apparent that the human isolates did not cluster according to the shared isolation source. Instead, strain MGYG-HGUT-00125 clustered together with cheese isolate FAM22552 in clade II, while the other four human isolates formed a separate cluster with Cirm BIA 2237 isolated from silage. This suggests that there might be certain similarities in the gene repertoire of these strains. Indeed, FAM22552 and MGYG-HGUT-00125 strains both contained a relatively high number of strain-specific genes, as shown later in this report. However, we could not find an explanation for co-clustering of human and silage isolates. Moreover, two mud isolates (i.e., 24853 and UBA5407) showed high similarity to silage isolate Cirm BIA 2237 in both trees, as did two milk isolates, namely Cl_82 and Cl_80, which clustered in close proximity in the pangenome-based tree. The close relationship between mud, silage and milk isolates offers a perspective on the origin and an acquisition of biological functions that will enable *C. tyrobutyricum* to survive or adapt various conditions such as high salt concentration and extreme pH or temperature. According to this perspective, several studies indicated that the main source of spores in clostridia is the natural environment. As a result, soil, silage and other feeds, and feces, are often contaminated with the spores, which can subsequently end up in manure or contaminate teats and thus facilitate their way to the raw milk (Vissers et al., 2006; Julien et al., 2008). It appears that the close relationship of the milk, mud and silage isolates may be explained by this main initial source of clostridial spores.

In both trees, strain Cl_52 clustered separately from all 27 *C. tyrobutyricum* strains and did not show similarity to other strains. This observation is consistent with the findings from the computed pairwise-comparisons, indicating that Cl_52 is distant from the other strains. The separate clustering in the pangenome tree was due to an abundance of unique genes in the genome of Cl_52 as further provided in the pangenome related section. In addition, the finding of the core-genome based phylogeny was fully supported by SNP phylogeny constructed by kSNP3 (Supplementary Figure S2). The distantly placed Cl_52 contained more SNPs (27,585) than other *C. tyrobutyricum* strains (0–966). It is not clear why some *C. tyrobutyricum* strains have more SNP variations than other strains, and what are the impacts of these SNP variations on the phenotype and function of these *C. tyrobutyricum* strains. More research is thus required to answer these questions.

Taken together, the results from phylogenetic analysis highlight the high level of genetic heterogeneity within the species *C. tyrobutyricum*. Our findings are consistent with those of previous studies (Bermudez et al., 2016; Burtscher et al., 2020; Podrzaj et al., 2020). For instance, Burtscher et al. (2020) found that most of the clusters, which encompassed more than two strains, consisted of a mixture of isolates from different sources, while some similarities were observed among strains from the same environment. It should be noted that previous studies almost exclusively investigated strains isolated from cheese and milk. To the best of our knowledge, this is the first large-scale phylogenetic analysis of *C. tyrobutyricum* comprising strains from different environments and isolation sources. By integrating the non-dairy isolates, the phylogenetic tree reconstructed here has broadened our knowledge of the phylogenetic relationship among *C. tyrobutyricum*. We hypothesize that the gain and the loss of different genes contributed to the differences in the genetic composition of *C. tyrobutyricum* strains and led to evolutionary divergence among *C. tyrobutyricum* strains. However, considering that most of the genomes used in this study are not closed, it is possible that due to errors in assembly, there may be risks of only detecting partial gene sequences, which may be misclassified as novel gene families, or not detecting certain genes at all (Schnoes et al., 2009; Denton et al., 2014; Perez-Cobas et al., 2020; Dida and Yi, 2021).

### Pan/Core Genome Analysis

Pangenome refers to a global gene repertoire of a species and is composed by the core genome, comprising the genes shared by all the analyzed strains, the accessory genome enclosing genes shared with two or more (but not all) strains, and the unique genome, which consists of strain-specific genes (Medini et al., 2005; Tettelin et al., 2008). To gain an overall approximation of the total gene pool for *C. tyrobutyricum*, the pangenome of the species was constructed on the basis of 28 *C. tyrobutyricum* genomes.

**Figure 3:**
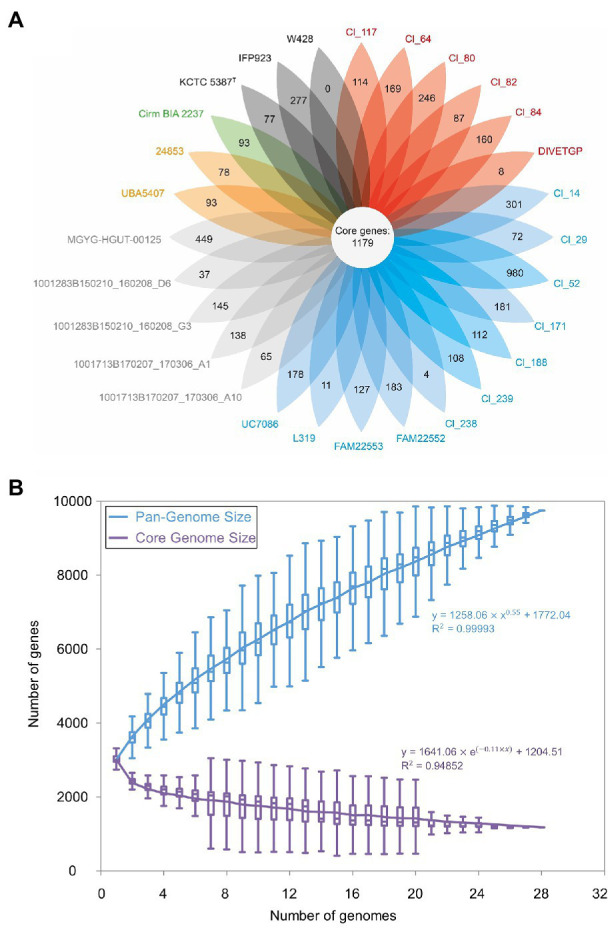
Genomic diversity of 28 *Clostridium tyrobutyricum* strains. **(A)** Core and strain-specific genes of 28 *C. tyrobutyricum* genomes. Each oval represents a strain. The number of genes shared by all strains (core genome) is in the center. The number of strain-specific genes is indicated in non-overlapping portions of each oval. The type strain is indicated by the superscript capital T. **(B)** Increase and decrease in gene families in the pan-(blue) and core (violet) genome, respectively. Gene accumulation curves were plotted as a function of the number of genomes sequentially added (*n* = 28) of the power-law regression model. The pangenome size is calculated at 9,748 genes. The trajectory of the pangenome shows characteristics of an open pangenome.

The size of the *C. tyrobutyricum* pangenome can be estimated to include 9,748 genes, which is found to be 3.14-fold of the average number of genes in each genome (3,113; Figure 3A). A total of 1,179 genes (12.1%) was shared by all strains constituting the core genome, while the remaining 8,569 (87.91%) were variably represented genes (Figure 3A). Although the core genome of the analyzed *C. tyrobutyricum* strains represented ~40% of the average number of genes per genome, when we consider it as a fraction of pangenome size, it is found to be relatively small (12.1%). By comparison, previous studies had found the *C. tyrobutyricum* core genome to contain 2,409–2,764 genes (Storari et al., 2016; Liu et al., 2020) representing roughly 80–96% of the total *C. tyrobutyricum* pangenome. This discrepancy is most probably due to the different number of genomes previously used (3–4) and the different methods employed to calculate core genes: in contrast to previous work, we have performed clustering of the 28 genomes and identified strain-specific genes within the merged dataset (see “Materials and Methods” section). The size of core genome of *C. tyrobutyricum* is found to be roughly proportional to the size of core genome of some other members of the genus *Clostridium* cluster I, including *Clostridium perfringens* [core genes = 1,020; *n* = 173 (Feng et al., 2020)] and *C. butyricum* [core genes = 1,011; *n* = 24 (Zou et al., 2021b)]. The presence of a small core genome in microbial species is not uncommon and has been observed in other species (McInerney et al., 2017). For instance, the analysis of 1,524 genomes of *Pseudomonas* revealed a core genome size of 3% of genes in pangenome, while the remaining 97% of genes represented the accessory genome (Karasov et al., 2018). In a study of 2,085 *Escherichia coli* genomes, 3,188 core genes represented a remarkably small fraction compared to the 90,000 genes comprising the *E. coli* pangenome, which is the largest pangenome analysis for single species to date (Land et al., 2015).

The contribution of every genome to the complete pangenome of *C. tyrobutyricum* is illustrated in Figure 3B, where the pangenome and core genome of the analyzed genomes are plotted. As can be seen, the size of the core genome converged and the pangenome size gradually expands without reaching a plateau with sequential addition of each genome considered. Furthermore, the fitted curve for the pangenome profile showed that the fitted exponent of the curve was positive, indicating that the pangenome of *C. tyrobutyricum* is open. This observation is in agreement with the few published reports on this topic on other members of the genus *Clostridium* cluster I (Udaondo et al., 2017), such as *Clostridium baratii* (Silva-Andrade et al., 2022), *C. perfringens* (Kiu et al., 2017; Feng et al., 2020), *C. beijerinckii* (Sedlar et al., 2021; Zou et al., 2021a), and *C. butyricum* (Zou et al., 2021b). Open pangenomes may indicate the necessity of constant genomic adaptations and diversification to cope with heterogeneous environments (Medini et al., 2005; Rouli et al., 2015). *Clostridium tyrobutyricum* is widely distributed in nature. This feature together with the evidence for an open pangenome suggests that the accessory genome of *C. tyrobutyricum* could confer specific phenotypes that are advantageous under certain selective conditions.

There were 4,081 gene clusters identified as partially shared genes comprising the accessory genome, which accounted for 41.87% of the pangenome. These genes are responsible for species diversity, environmental adaptation and other characteristics of bacteria. In addition, strain-specific genes represented 46.04% (4,488) of the total pangenome (Figure 3A). Notably, the distribution of strain-specific genes was diverse, varying from 4 to 980 proteins, in which the strain Cl_52 clearly stood out by possessing the highest number of strain-specific genes (980), whereas *C. tyrobutyricum* strain W428 did not present strain-specific genes at all (Figure 3A). The fact that W428 derived from the type strain may explain lack of unique set of genes in this strain.

The heatmaps generated based on the matrix of presence or absence of genes in *C. tyrobutyricum* pangenomes also displayed the variation in the distribution of accessory genes among strains (Supplementary Figure S3). The majority of the gene clusters specific for individual strains encoded hypothetical proteins with unknown functions. These unique genes might be related to extraordinary phenotypes observed previously (Podrzaj et al., 2020) and separate clustering in all phylogenetic analyses. However, the specific genes and pathways conferring the phenotypic variation remain to be identified. Besides, Cl_52 clearly stood out due to the absence of a large number of genes (483 genes). This observation again supported the results from phylogenetic and phylogenomic analyses.

The notable difference of the unique genes between some strains suggested that these strains may have expanded in various habitats and that these genes may have been gained *via* horizontal gene transfer (HGT) or differential gene loss, which are considered as the most common ways to acquire new genes (Arnold et al., 2021), or even gene duplications, followed by diversification (Bobay and Ochman, 2018). Horizontal movement of genes is believed to be important for biological properties, which are beneficial to the host organisms because they enable changes in the metabolic and nutritional capacities and lead to adaptation to more dynamic habitats. Several genomic studies on *C. butyricum*, *C. perfringens*, and *Clostridium botulinum* have demonstrated that some elements related to antibiotic resistances genes, toxin genes and virulence genes have been a result of HGT between and within *Clostridium* species (Popoff and Bouvet, 2013; Lacey et al., 2017). To date, no specific genes acquired *via* HTGs have been detected in *C. tyrobutyricum*.

Noteworthily, it is clear that pangenome evaluations strongly depend upon both the number of genomes and the quality of genome sequences. As indicated, the dataset used in this study (28 genomes) was not large enough to define a total accessory genome of *C. tyrobutyricum.* We speculate that the addition of more high-quality genomes and genomes from strains of different origins would probably continue to increase the size of the accessory gene set. Thus, we expect that due to the widespread distribution of *C. tyrobutyricum*, additional genetic variation potentially exists.

### Functional Assignment of *Clostridium tyrobutyricum* Pangenome Fractions

**Figure 4:**
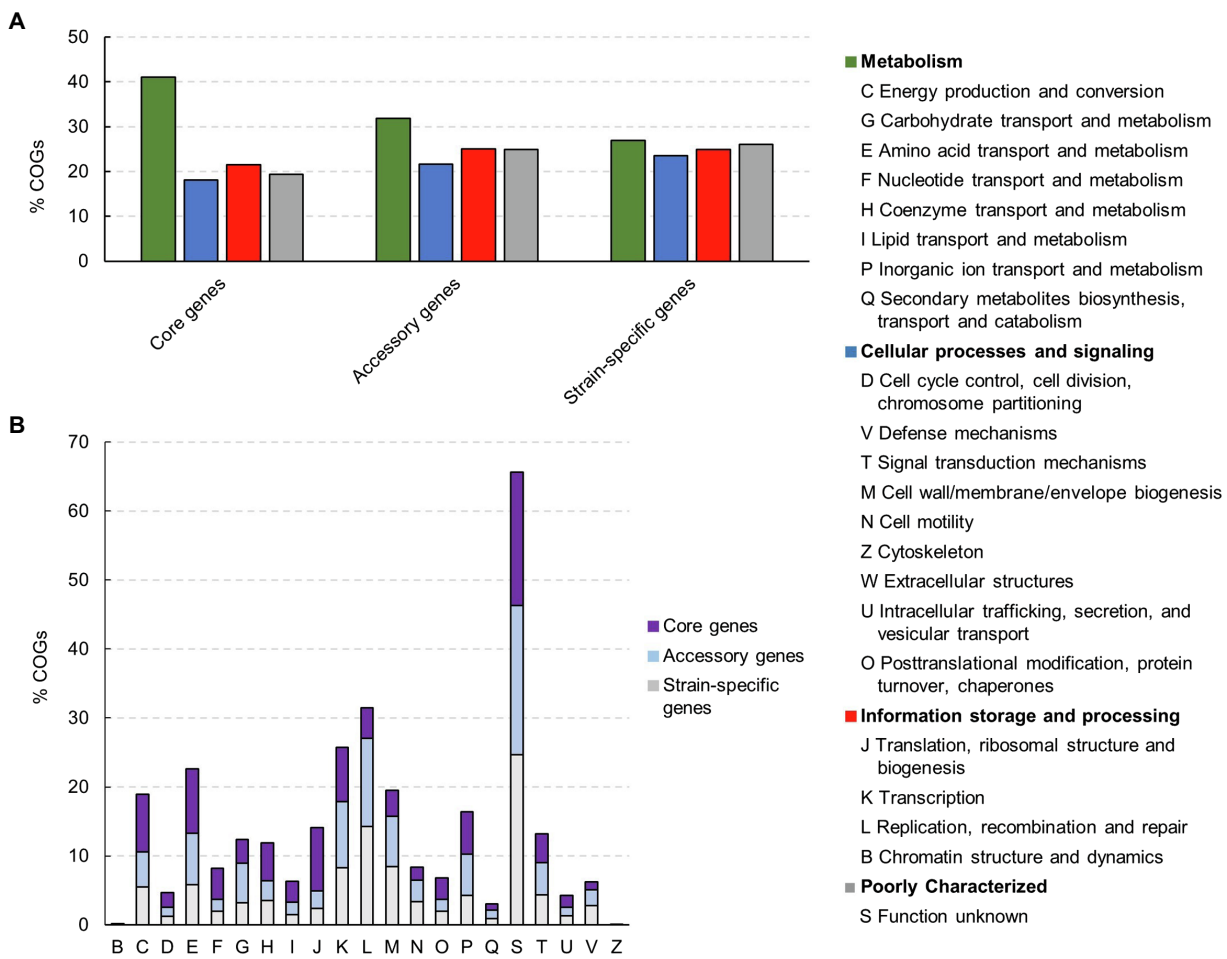
Clusters of orthologous groups (COGs) in core- and accessory genome, and strain-specific genes and their associated functions in 28 *Clostridium tyrobutyricum* genomes. **(A)** Distribution of functional COGs in each core, accessory, and unique genome. **(B)** Detailed distribution of COGs with their functions.

An open state of bacterial pangenome reflects the diversity within the gene pool of a given species. In order to investigate the functional composition and diversity of proteins encoded by the core, accessory and strain-specific genes of *C. tyrobutyricum*, we performed Clusters of Orthologous Groups (COGs) and KEGG analysis (Figure 4).

As expected, the core genome was enriched with genes related to fundamental roles in the maintenance of basic cellular processes. The housekeeping COG categories included “amino acids transport and metabolism” (9.33%) “inorganic ion transport and metabolism” (6.14%), “energy production and conversion” (8.32%), and “coenzyme transport and metabolism” (5.46%). High abundance of these gene categories within the core genome indicates that the strains are metabolically similar, which is consistent with other clostridia, including *C. butyricum*, *C. beijerinckii*, *C. sporogenes*, and *C. baratii*, which have open pangenomes (Wu et al., 2017; Sedlar et al., 2021; Zou et al., 2021b; Silva-Andrade et al., 2022). Furthermore, genes from the category “translation, ribosomal structure and biogenesis” were also abundant in the core genome (1.59%). This is not surprising due to the fact that the translation machinery is universal and essential to cellular life, and thus expected to dominate the set of genes that are shared by all strains of a given species (Bowman et al., 2020). As core genes should be present in every strain, they could be utilized as the candidates for reference genes for reverse transcription quantitative PCR (RT-qPCR) in *C. tyrobutyricum*.

On the contrary, accessory and strain-specific genes both contained higher proportions of genes related to “information storage and processing” compared to the core genome. In particular, the accessory genome was enriched with proteins from the category “transcription,” such as transcriptional regulators belonging to various families including Lysr, DeoR, LacI, AraC, Fis, Lrp/AsnC, and MarR. Regulatory proteins play a major role in bacterial responses to specific environmental and cellular signals that modulate transcription, translation or other events related to gene expression. Such adaptive response can help to elucidate bacterial survival in unstable environments (Romero-Rodriguez et al., 2015).

Bacteria have evolved a number of mechanisms for coping with stress and adapting to changing environmental conditions. Many bacteria respond to a rapid temperature downshift (cold shock) by the production of small cold shock proteins (Csp; Graumann and Marahiel, 1996; Beales, 2004). During cold shock, the cell membrane fluidity and enzyme activity decrease, and the efficiency of transcription and translation is reduced due to stabilization of nucleic acid secondary structures. Moreover, protein folding is inefficient and ribosome function is hampered. Csps are thought to counteract these harmful effects by serving as nucleic acid chaperons that may prevent the formation of secondary structures in mRNA at low temperature and thus help cells to adapt (Phadtare, 2004). Interestingly, although Csps (e.g., *cspsA*, *cspsL*, Cold shock protein 2) were identified in all strains, additional genes for *cspsA* and *cspsL* were harbored by few cheese isolates (i.e., Cl_238, Cl_239, and Cl_52) and one raw milk isolate (i.e., Cl_80). Considering the results from our previous study in an experimental cheese broth, both Cl_80 and Cl_238 were among the strains that were able to produce gas at a low temperature (14°C). Furthermore, given that strain Cl_238 shared the same isolation source with Cl_239 and these two strains showed exceptionally high similarities based on whole-genome comparison (Supplementary Tables S2–S4), we speculate that similar physiological properties may be expected from these two isolates. In contrast, no additional Csps genes were found in isolates Cl_64 and Cl_84, that also showed growth at low temperatures. Previous studies on *C. botulinum* demonstrated that Csps also contribute to osmotic, pH and ethanol stress tolerance (Derman et al., 2015). To the best of our knowledge, no work has been focused on *C. tyrobutyricum* in this regard.

Another response to temperature changes by bacteria is the maintenance of an adequate liquid-crystalline balance by changing the membrane lipid composition. Among the identified genes, phophatidyltransferases are involved in phoshoplipid biosynthesis, and protein phophatidylglycerol lysyltransferase encoding gene *mprF* catalyzes the transfer of a lysyl group from L-lysyl-tRNA(Lys) to membrane-bound phosphatidylglycerol (PG), which produces lysylphosphatidylglycerol (LPG), a major component of the bacterial membrane with a positive net charge, and therefore plays an important role in bacterial virulence (Peschel et al., 2001). It has been reported that microorganisms, such as *E. coli* (Sinensky, 1974; Jones et al., 1987) and *Bacillus subtilis* (Svobodova et al., 1988; Suutari and Laakso, 1992; Beranova et al., 2008), were able to regulate membrane lipid composition to increase their tolerance to low temperatures. Recently, Zhu and Yang (2003) demonstrated that a *C. tyrobutyricum* strain adapted to higher butyrate concentrations contained increased concentrations of saturated fatty acids (C16:0 and C17:0) in its cell membrane. Such enrichment could result in a better maintenance of a microorganism’s biological functions under low/high temperature.

In terms of osmoregulation, potassium (K^+^) ions are crucial for bacteria to regulate solute transport and adapt to changes in osmotic pressure. There are various systems for accumulation of K^+^ ions in the cell, including Kup, Trk, Kdp, and Ktr transport systems (Diskowski et al., 2015). The KdpD-KdpE transporter system was found in several strains (e.g., Cl_14, FAM22552, Cl_188, and Cl_117) and is a suggestive of a strategy to survive under relatively high osmolality. Another defense mechanism is the accumulation of osmoprotectants, such as ectoine, proline, and glycine betaine in the cytoplasm. Indeed, six different betaine/carnitine/choline family transporters have been identified in strains isolated from cheese (i.e., Cl_238, Cl_239, and UC7086), whereas a lower number (3–4) was found in other *C. tyrobutyricum* strains.

Interestingly, cheese and milk isolates contained a large proportion of genes involved in “post-translational modification, protein turnover, and chaperones” that may account for survival under stressful environmental conditions during cheese ripening. Of the cheese and milk isolates, 15% encode proteins associated with membrane transport, such as permease and ABC-transporters, suggesting exchange between the strain and its environment. Furthermore, we identified several genes that seem to be specifically associated with cheese and milk isolates and are linked to transcriptional regulation, suggesting that these regulators act on specific genes to control their expression and confer an advantage when present in cheese. It would be interesting to study which genes are affected by these transcriptional regulators to assess the mechanisms employed to survive under cheese ripening conditions such as low pH, low water activity and high salt concentration.

As butyric acid producing clostridia, *C. tyrobutyricum* can ferment lactic acid into butyric acid, acetic acid and gas (CO_2_ and H_2_) from metabolism of lactose (Drouin and Lafrenière, 2012). Although genes related to “carbohydrate metabolism and transport” were enriched in accessory genome, genes encoding enzymes required for fermenting hexoses through to pyruvate *via* an Embden-Mayerhof-Parnas pathway were present in all *C. tyrobutyricum* genomes. Among them, genes encoding fructose-1.6-biphosphate aldolase (*fbp*), acetate kinase (*ack*), phosphotransacetylase (*pta*), methylglyoxal synthase (*mgsA*), hydroxyacylglutathione hydrolase (*gloB*), lactate racemase (*larA*), and L-lactate dehydrogenase (*ldh*), which plays a key role in the production of L-lactate from pyruvate (Jiang et al., 2018; Detman et al., 2019), were identified as core genes, but the copy number of some genes varied among strains. This observation is particularly interesting because previous studies have reported differences in gas production among *C. tyrobutyricum* strains (Garde et al., 2012b; Ruusunen et al., 2012; Silvetti et al., 2018; Podrzaj et al., 2020). For instance, the type strain and FAM22552 contained multiple copies of *fbp*, Indeed, this strain (Cl_20 in Podrzaj et al., 2020) showed faster gas production in experimental cheese broth at 37°C compared to other tested strains (Podrzaj et al., 2020). Although it is plausible that late blowing defect in cheese by some strains could have been a consequence of high number of carbohydrates metabolizing genes, in several cases the present annotation of genes did not agree with the observed phenotype. For instance, strain Cl_52 contained besides above-mentioned genes, multiple copies of gene encoding hydroxyacylglutathione hydrolase, yet, no metabolic activity was observed at any of the tested conditions in phenotypic assays (Podrzaj et al., 2020). However, the causality of correlations between the identified genes and the observed phenotypes has not been proven yet. Additional experiments such as knocking out target genes, or over-expressing gene in strains that lack phenotype, will increase the understanding of the type of relation between the target gene and phenotype.

Moreover, a large proportion of strain-specific genes (23.55%) was related to the category “cellular processes and signaling.” In particular, “cell wall/membrane biogenesis” (8.37%) and “defense mechanisms” (2.76%) were higher in number compared to accessory genes (2.32 and 7.33%, respectively). It is worth noticing, for instance, that ABC-type transport system components were found in strain-specific genes. This observation concurred well with the concept that the accessory or unique fraction of any genome usually contain non-essential and/or regulatory genes, especially the ones that would facilitate the adaptation of the respective species to any specific environment or lifestyle.

Finally, genes with “unknown functions” were enriched in all three pangenome fractions (core, accessory, and strain-specific genes), indicating that there are still many features of *C. tyrobutyricum* that remain unreported. The over-representation of “function unknown” in the COG categories was similar to what has been observed in other studies of *Clostridium* species including *C. beijerinckii*, *C. butyricum*, and *C. ljungdahlii* (Philips et al., 2017; Sedlar et al., 2021; Zou et al., 2021a).

According to the KEGG database, categories amino acid, nucleotide, energy and cofactors, and vitamins metabolisms were highly conserved functions among *C. tyrobutyricum* strains, while signal transport was encoded mostly by accessory and strain-specific genes (Supplementary Figure S4). The membrane transport, cellular communication and signal transport genes belonged to accessory or unique fractions of the pangenome. Corroborating COGs data, replication and repair functions of *C. tyrobutyricum* were mostly located in strain-specific genes, which could be an indicative of increased recombination. This can be attributed to the fact that the strains originated from diverse isolation sources and therefore possessed a larger number of accessory genes for maintaining replication and cell division in various environments.

Additionally, we found that the genomes of 21 *C. tyrobutyricum* strains were enriched in CRISPR elements. The CRISPR system is an RNA-mediated adaptive immunity system that plays critical role in defense against invasive genetic elements such as phages and plasmids (Barrangou et al., 2007; Marraffini and Sontheimer, 2008). Interestingly, the prevalence of CRISPR systems in *C. tyrobutyricum* is relatively high (64.3% strains). CRISPR systems were less common in strains isolated from raw milk (33.3%) compared to those from human, cheese, and mud isolates (100, 54.5, and 50%, respectively; Supplementary Table S7). Among the strains containing CRISPR-Cas systems, we found a series of up to seven Cas genes: *cas1*, *cas2*, *cas3*, *cas4*, *cas5*, *cas6*, and *cas7* (*Csh2* family), which is in concordance with a previous study (Wu et al., 2017). Strains with the largest number of Cas genes were the type strain, L319 and W428 (19 genes), followed by strain Cirm BIA 2237 (18 genes) and strain 24853 (15 genes). It has been suggested, that strains with higher numbers of these genes may be more prone to exchange genetic material with other strains and hence evolution (Hatoum-Aslan and Marraffini, 2014).

## Conclusion

In summary, this is the first and the most comprehensive comparative genome analysis of *C. tyrobutyricum* to date. Our results indicate that *C. tyrobutyricum* strains are highly diverse. The *C. tyrobutyricum* pangenome was observed to be in an open state, which might account for the observed genomic variability. However, core-gene and pangenome based phylogenetic analyses showed that dairy-related strains tend to cluster together, suggesting higher similarity in their genetic content. However, many genes in the accessory genome encode proteins of yet unknown function. This feature together with the fact that genes can be inactivated or differentially expressed, means that many unique properties of the species *C. tyrobutyricum* might be still unreported. The characterized divergences and similarities among strains represent a notable contribution towards understanding the diversity in spoilage potential of *C. tyrobutyricum* and provide reference value for further studies exploring the gene modules responsible for the spoilage ability of this species. With the generation of additional complete chromosomes of *C. tyrobutyricum* strains related to late blowing, insights into gene acquisition, deletion and maintenances will facilitate a better understanding of the genetic backgrounds of cheese spoilage caused by *C. tyrobutyricum*. Future molecular and phenotypic studies are encouraged to characterize the functional roles for the unknown genes.

## Data Availability Statement

The original contributions presented in the study are included in the article/**Supplementary Material**, further inquiries can be directed to the corresponding author.

## Author Contributions

JB, KD, and LP designed the study. LP performed the bioinformatics analyses, analyzed the data, and wrote the original draft of the manuscript. JB and KD reviewed and edited the manuscript. All authors contributed to the article and approved the submitted version.

## Conflict of Interest

The authors declare that the research was conducted in the absence of any commercial or financial relationships that could be construed as a potential conflict of interest.

## Publisher’s Note

All claims expressed in this article are solely those of the authors and do not necessarily represent those of their affiliated organizations, or those of the publisher, the editors and the reviewers. Any product that may be evaluated in this article, or claim that may be made by its manufacturer, is not guaranteed or endorsed by the publisher.

## Footnotes

^1^ https://www.ncbi.nlm.nih.gov/genbank/

^2^ https://www.ncbi.nlm.nih.gov/biosample/

^3^ https://crisprcas.i2bc.paris-saclay.fr/CrisprCasMeta/Index

^4^ https://github.com/widdowquinn/pyani

^5^ http://ggdc.dsmz.de/

^6^ http://sanger-pathogens.github.io/Roary

^7^ http://www.ezbiocloud.net
