# Supplementary material for: Comparative Genomics Provides Insights Into Genetic Diversity of Clostridium tyrobutyricum and Potential Implications for Late Blowing Defects in Cheese
Source: Front Microbiol. 2022 Jun 2;13:889551. doi: 10.3389/fmicb.2022.889551 (PMC9201417; doi:10.3389/fmicb.2022.889551)
Supplement: Supplementary file 1 [file Data_Sheet_1.DOCX]

Supplementary Figures

Comparative genomics provides insights into genetic diversity of *Clostridium tyrobutyricum* associated with late blowing defects in cheese

Lucija Podrzaj^1^, Johanna Burtscher^1*^, Konrad J. Domig^1^

^1^ University of Natural Resources and Life Sciences, Vienna, Department of Food Science and Technology, Institute of Food Science, Muthgasse 18, 1190 Vienna, Austria

***** Correspondence:
Johanna Burtscher
[johanna.burtscher@boku.ac.at](mailto:johanna.burtscher@boku.ac.at)

**
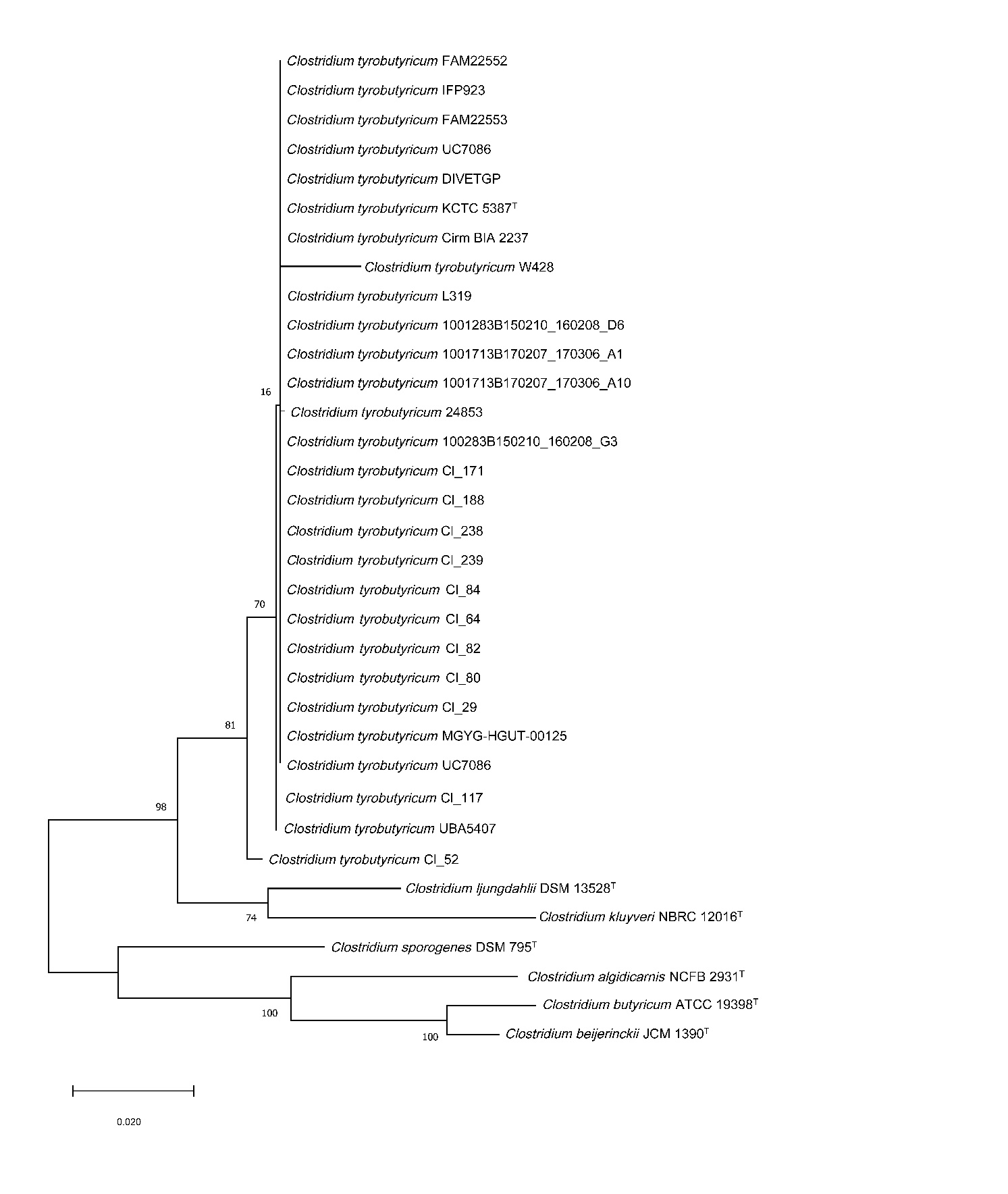
**

**Supplementary Figure S1.** Phylogenetic tree constructed using maximum-likelihood method (ML) and Kimura 2-parameter method (Kimura, 1980) based on the 16S rRNA gene sequence of 28 *Clostridium tyrobutyricum* strains and their close relative type strains within the genus *Clostridium*. Bootstrap values are expressed as percentages of 500 replicates. Bar indicates 0.02 substitutions per nucleotide position. The type strain is indicated by the superscript capital T.





^T^

**Supplementary Figure S2.** A maximum-likelihood (ML) tree based on core SNP analysis of 28 *Clostridium tyrobutyricum* strains using kSNP3 (Gardner et al., 2015). The numbers in the nodes denote bootstrap values. The type strain is indicated by the superscript capital T. Bar indicates 0.05 substitutions per site.


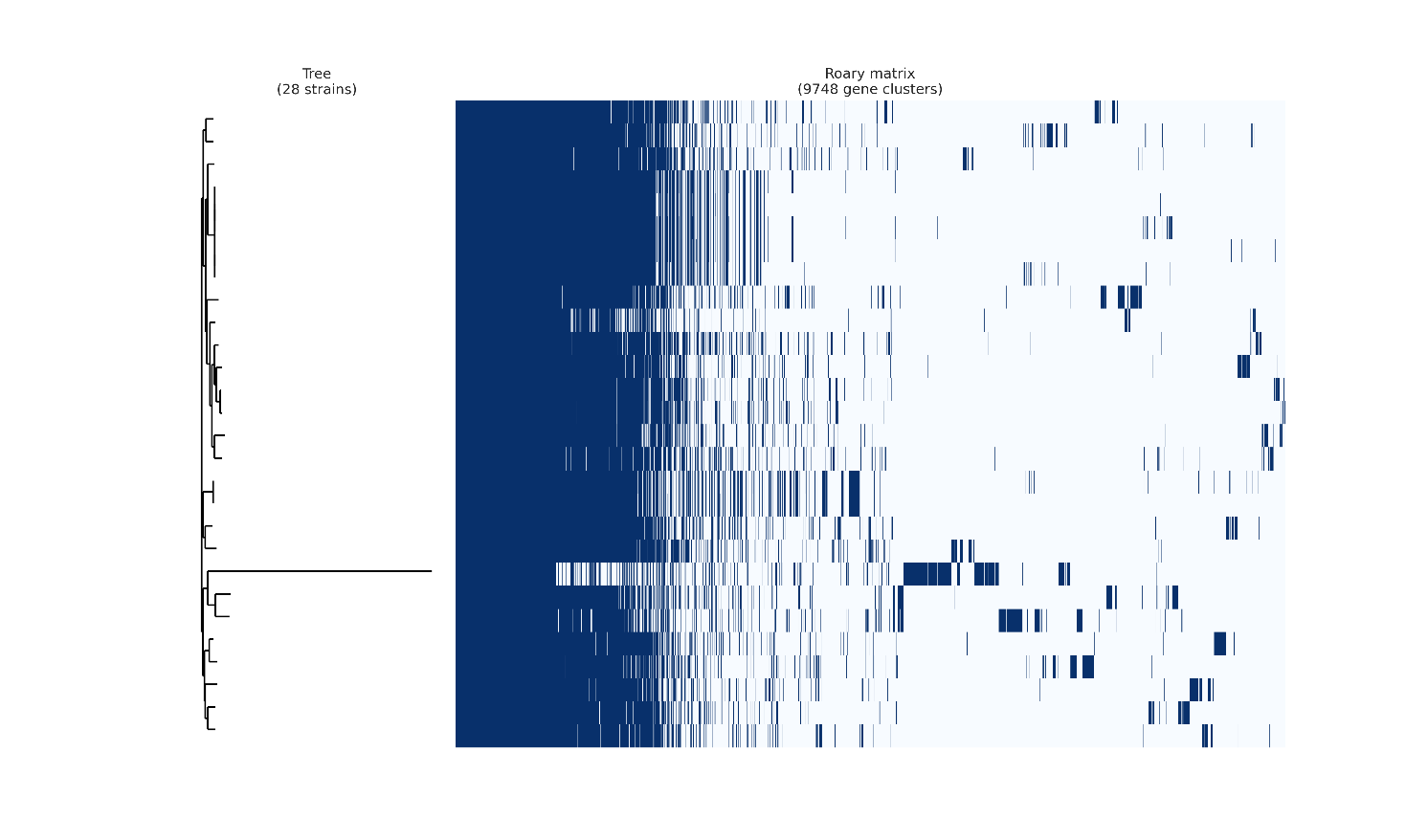

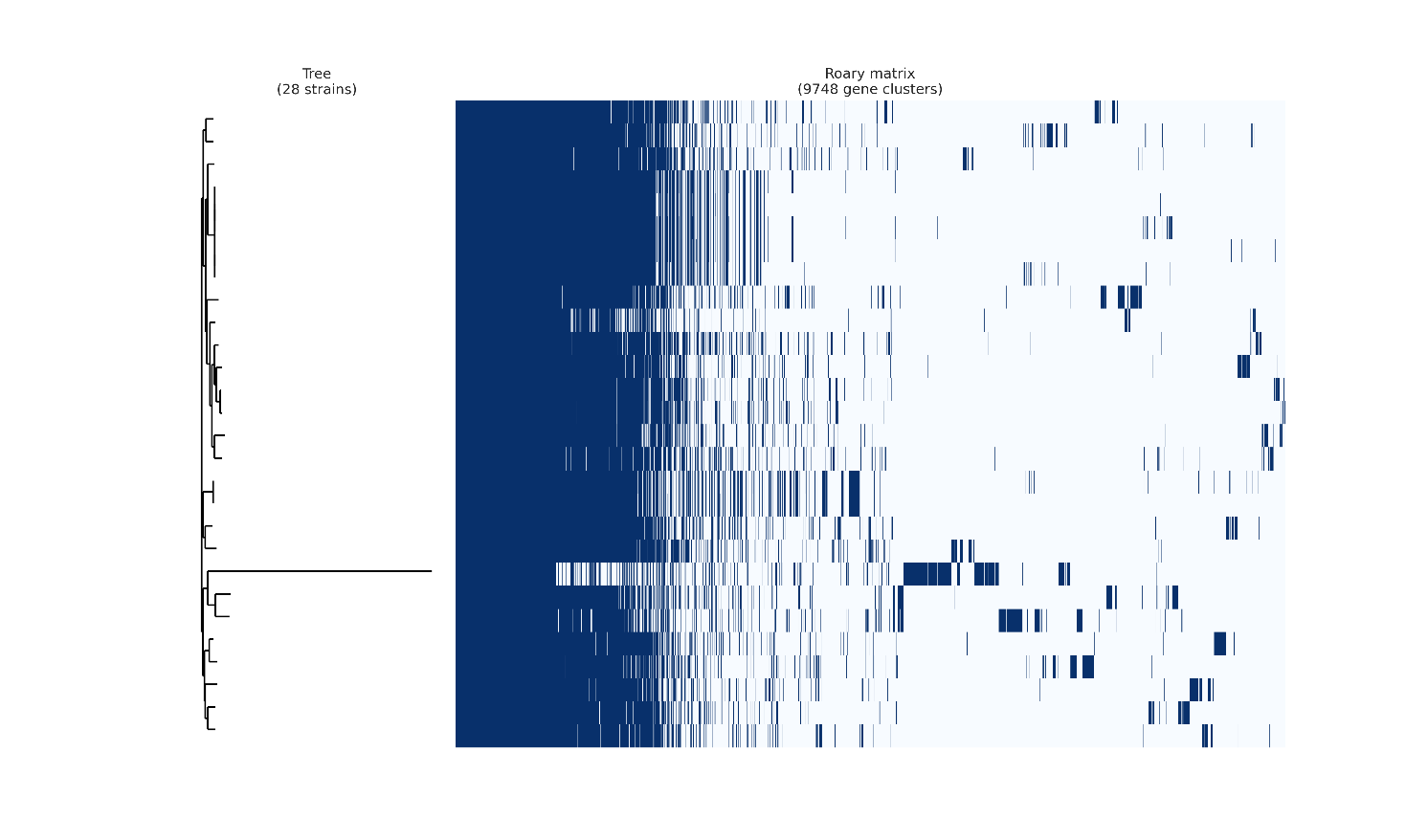


FAM22553

Cl_80

Cl_82

W428

DIVETGP

KCTC 5387^T^

L319

Cl_29

IFP923

UBA5407

Cirm BIA 2237

1001713B170207_170306_A1

1001713B170207_170306_A10

1001283B150210_160208_G3

1001283B150210_160208_D6

24853

Cl_239

Cl_238

Cl_117

Cl_64

Cl_52

FAM22552

MGYG-HGUT-00125

Cl_84

Cl_14

Cl_171

UC7086

Cl_188

**Supplementary Figure S3.** Phylogenetic reconstruction and heatmap based on the matrix of presence or absence of genes in the *Clostridium tyrobutyricum* pangenome. The tree was constructed by the FastTree tool using the Roary pipeline. Each row corresponds to a branch on the tree. Each column represents an orthologous gene family. Dark columns of the heatmap indicate the presence of a gene, while light colors indicate the absence of genes. The type strain is indicated by the superscript capital T.

**Supplementary Figure S4.** Functional distribution of core, accessory, and strain-specific genes in the pangenome of *Clostridium tyrobutyricum* according to KEGG categories. Core, accessory and strain-specific genes are indicated in violet, blue and gray, respectively.

**References**

Gardner, S.N., Slezak, T., and Hall, B.G. (2015). kSNP3.0: SNP detection and phylogenetic analysis of genomes without genome alignment or reference genome. *Bioinformatics* 31(17)**,** 2877-2878. doi: 10.1093/bioinformatics/btv271.

Kimura, M. (1980). A simple method for estimating evolutionary rates of base substitutions through comparative studies of nucleotide sequences. *J Mol Evol* 16(2)**,** 111-120. doi: 10.1007/BF01731581.
